# Feasibility and safety of watershed detection by contrast-enhanced ultrasound in patients receiving peripheral venoarterial extracorporeal membrane oxygenation: a prospective observational study

**DOI:** 10.1186/s13054-020-02849-y

**Published:** 2020-04-02

**Authors:** Nina Buchtele, Thomas Staudinger, Michael Schwameis, Christian Schörgenhofer, Harald Herkner, Alexander Hermann, UltraECMO investigators, Gerhard Ruzicka, Christoph Weiser, Hans Domanovits, Alexander O. Spiel, Peter Schellongowski, Andja Bojic, Anne Merrelaar, Monika Schmid

**Affiliations:** 1 Department of Medicine I, Intensive Care Unit 13i2, Medical University of Vienna, Vienna, Austria; 2 Department of Emergency Medicine, Medical University of Vienna, Währinger Gürtel 18-20, 1090 Vienna, Austria; 3 Department of Clinical Pharmacology, Medical University of Vienna, Vienna, Austria

**Keywords:** Extracorporeal membrane oxygenation, Contrast media, Ultrasonography, Feasibility studies

## Background

In bifemoral venoarterial extracorporeal membrane oxygenation (VA ECMO), the transition point at which the antegrade pulsatile output from the left ventricle and the retrograde non-pulsatile ECMO output collide is referred to as watershed [1]. Currently, no standard method is available to determine its location. Occasionally, contrast-enhanced computed tomography (CT) or angiography has been used [1–3]. Both techniques, however, bear disadvantages including radiation exposure and use of iodinated contrast media. We assessed the feasibility and safety of contrast-enhanced ultrasound (CEUS) to detect the watershed at the bedside in patients on bifemoral VA ECMO at three ICUs of a European tertiary care facility.

## Methods

CEUS was performed as soon as possible after ECMO-initiation (Cardiohelp, Maquet, Germany) using SonoVue contrast media (Bracco, Italy). Transesophageal echocardiography (x7-2t probe) and transabdominal sonography (3–5 MHz curvilinear probe) were performed concomitantly to display mid-esophageal aortic valve, ascending, descending aorta, and upper esophageal aortic arch long-axis views as well as longitudinal views of the proximal (below diaphragm), mid (level of renal arteries), and distal (above iliac bifurcation) abdominal aorta. The mechanical index was set to 0.05–0.10 field of view. Prior to CEUS, the arterial bubble sensor activating zero-flow mode was disabled. The acoustic alarm was kept active. The presence or absence of pulsatility in the left radial artery was documented.

One milliliter of SonoVue was administered via the venous drainage cannula, followed by a flush of 10 ml normal saline. The obtained images were evaluated qualitatively. If a watershed area was not able to be visualized, contrast-enhanced blood flow was classified into “pulsatile” or “continuous” to discriminate between cardiac and ECMO blood flow. The feasibility of CEUS was assessed based on qualitative image evaluation, the amount of contrast media administered, and the rate of bubble detection. Secondary outcomes were safety and frequency of radial arterial pulsatility. Safety variables included ECMO settings, hemodynamics, and neurologic assessment and were obtained over a 6-h period after CEUS.

The variables are presented as absolute values (*n*), relative frequencies (%), and median (25–75% IQR). We used random-effects general linear regression models to estimate mean changes for each safety variable (mean ± SD).

## Results

**Fig. 1:**
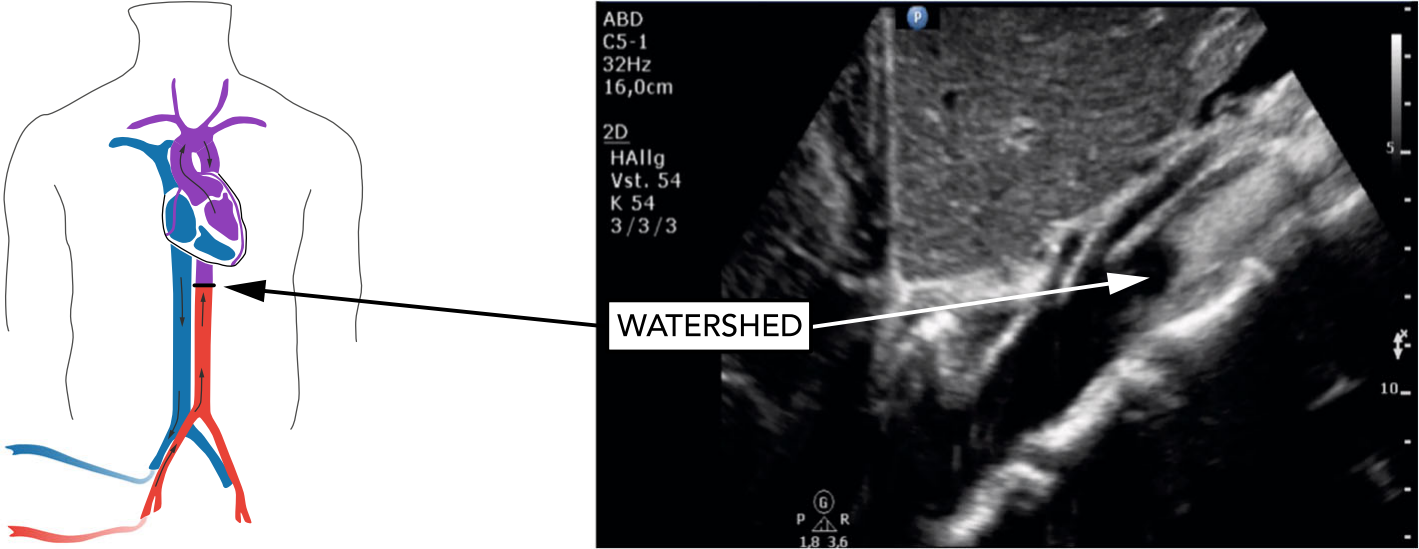
Visualization of contrast-enhanced retrograde non-pulsatile ECMO blood flow. The watershed is marked with an arrow and located distal to the superior mesenteric artery

Between August 2018 and April 2019, ten patients were enrolled (Table 1). Qualitative detection of watershed location by CEUS was feasible using 1 ml contrast media. In five patients, the watershed could be clearly shown in the abdominal aorta, seconds after contrast media administration (Fig. 1). In the remaining five patients, contrast-enhanced continuous blood flow was visible throughout the abdominal and thoracic aorta indicating watershed location close to the aortic root. The pulsatility of the left radial arterial waveform and opening of the aortic valve was present in all patients. Acoustic bubble detection occurred in all patients after CEUS. No changes in the safety variables related to CEUS occurred (Table 1). CT imaging of the brain (8/10 patients) showed no cerebral lesions suggesting particle embolism.

**Table 1:** Baseline characteristics and changes of safety variables from baseline to 6 h after contrast media application

**Baseline characteristics**					
Male sex			9 [90]		
Age (years)			52 (50–55)		
Reason for ECMO					
Refractory cardiac arrest			6 [60]		
Cardiogenic shock			4 [40]		
Presence of severely impaired left ventricular systolic function			10 [100]		
Time from ECMO start to study inclusion (days), min to max			0–1		
Successful ECMO weaning			5 [50]		
Good neurologic outcome (CPC 1–2)			3 [30]		
Post-oxygenator paO_2_ after contrast-enhanced ultrasound (mmHg)			489 (439–507)		
**Safety parameters**					
**Variable**	**Baseline (*n*** **= 10)**	**5 min (*n*** **= 10)**	**15 min (*n*** **= 10)**	**2 h (*n*** **= 9)**	**6 h (*n*** **= 8)**
Ventilator FiO_2_ (%)	48 (40–80)	48 (40–80)	48 (40–80)	55 (45–80)	48 (40–70)
Peak pressure (mbar)	19.5 (18–21)	19 (18–21)	19 (18–21)	20 (19–23)	18 (18–23)
MAP (mmHg)	73 (70–81)	73 (64–93)	73 (64–93)	77 (73–82)	82 (74–94)
Norepinephrine (μg/kg/min)	0.34 (0.209–0.670)	0.250 (0.148–0.480)	0.250 (0.148–0.480)	0.420 (0.190–0.620)	0.410 (0.142–0.715)
Heart rate (bpm)	97 (66–111)	91 (64–107)	90 (64–107)	80 (61–105)	95 (72–107)
Pump speed (rpm)	2776 (2530–3151)	2698 (2530–3000)	2698 (2530–3000)	2741 (2530–3000)	3050 (2590–3300)
Blood flow (l/min)	2.23 (1.61–2.90)	2.13 (1.58–2.63)	2.13 (1.58–2.63)	2.37 (1.70–2.80)	2.76 (2.10–3.31)
New-onset pupil dilation and/or anisocoria	0	0	0	0	0
	**SD overall**	**SD within**	**Mean change (95% confidence interval)**		*p*value*
Ventilator FiO_2_ (%)	23.72	8.59	− 3.39 (− 7.54 to 0.75)		0.109
Peak pressure (mbar)	3.71	1.11	0.44 (− 0.63 to 1.51)		0.420
MAP (mmHg)	13.32	8.31	− 1.03 (− 8.31 to 6.25)		0.781
Norepinephrine (μg/kg/min)	0.32	0.15	− 0.23 (− 0.18 to 0.13)		0.774
Heart rate (bpm)	25.84	5.68	− 0.87 (− 5.68 to 3.93)		0.721
Pump speed (rpm)	1305.51	159.90	0.67 (− 86.46 to 87.80)		0.988
Blood flow (l/min)	0.84	0.41	0.073 (− 0.15 to 0.29)		0.512

Data are *n* [%] or median (25–75% IQR), unless indicated otherwise. For the random-effects general linear regression models, means with standard deviations were calculated. During the observation period, no changes in hemodynamic values, vasopressor dose, respirator, and ECMO settings occurred in temporal relationship to SonoVue administration. Two patients had already dilated pupils at the time of ultrasound study. Both patients died during the 6-h observation period following a decision to withdraw treatment by the treating physician

*CPC* cerebral performance category, *ECMO* extracorporeal membrane oxygenation, *FiO*_*2*_ fraction of inspired oxygen, *MAP* mean arterial pressure, *SD* standard deviation

*Results from random-effects general linear regression model

## Discussion

This study assessed the feasibility of CEUS for watershed detection at the bedside in patients on bifemoral VA ECMO. CEUS was apparently safe and provided real-time assessment of the watershed or contrast-enhanced continuous blood flow in the aorta. Increasing evidence indicates that CEUS is safe in critically ill patients, and application areas are ever-expanding [4–6]. In bifemoral VA ECMO, CEUS may help to identify patients at risk for differential hypoxia, given that left radial arterial pulsatility was present in all study patients, including those in whom the watershed was located near the aortic root.

## Limitations

Transthoracic suprasternal echocardiography may be useful to localize the watershed in the aortic arch but has not been tested. Furthermore, no reference imaging technique has been used to assess the performance of CEUS, because no standard method for the detection of the watershed is available, and no repeated measurements were performed.

## Data Availability

The datasets analyzed during the current study are available from the corresponding author on reasonable request. Complete results from safety data are available with the main manuscript. Exemplary ultrasound loops are available from the corresponding author on request.

## Abbreviations

CEUS: Contrast-enhanced ultrasound
CT: Computed tomography
VA ECMO: Venoarterial extracorporeal membrane oxygenation
